# Deciphering the shared genetic architecture between female reproductive disorders and psychiatric disorders

**DOI:** 10.1186/s13048-026-01970-w

**Published:** 2026-01-23

**Authors:** Nijie Li, Youhua Chen, Weie Zhao, Manchao Li, Yujie Li, Cong Fang, Panyu Chen

**Affiliations:** 1 Department of Reproductive Medicine Center, The Sixth Affiliated Hospital of Sun Yat- sen University, Guangzhou, Guangdong People’s Republic of China; 2 Guangdong Engineering Technology Research Center of Fertility Preservation, Guangzhou, Guangdong, People’s Republic of China; 3 Biomedical Innovation Center, The Sixth Affiliated Hospital, Sun Yat-sen University, Guangzhou, Guangdong, People’s Republic of China; 4 School of Nursing, Jinan University, Guangzhou, Guangdong, People’s Republic of China

## Abstract

**Background:**

The clinical association between female reproductive disorders, such as endometriosis, polycystic ovary syndrome(PCOS), uterine leiomyoma, and female infertility and psychiatric disorders, such as major depressive disorder(MDD), schizophrenia, and anxiety disorders has been widely reported. However, the genetic mechanisms underlying their comorbidity remain unclear. This study aimed to elucidate the genetic links between these disease categories through comprehensive genomic analyses.

**Methods:**

We analyzed genome-wide association study data from the Psychiatric Genomics Consortium and FinnGen database. Genetic correlations were estimated using linkage disequilibrium score regression and high-definition likelihood methods. Cross-trait meta-analyses through Multi-Trait Analysis of Genome-Wide Association Studies and Cross-Phenotype Association Analysis identified pleiotropic loci, followed by Fine-mapping with the ANNOVAR tool. Gene-based analyses integrated summary-data-based Mendelian randomization, multi-marker analysis of genomic annotation, and genome-wide complex trait analysis-fast gene-based association test approaches. Bidirectional Mendelian randomization assessed causal relationships using several complementary methods.

**Results:**

We identified significant genetic correlations between endometriosis and Attention-Deficit/Hyperactivity Disorder, Bipolar disorder(BD), and MDD, as well as between infertility/PCOS and MDD. Cross-trait analyses pinpointed five shared loci, with fine-mapping supporting their role as credible causal variants. Gene annotation implicated specific candidate genes, including *ARL14EP* for the endometriosis-BD link, which was further validated across SMR, MAGMA, and GCTAfastBAT analyses. Mendelian randomization demonstrated a causal effect of MDD on the risk of both endometriosis and infertility.

**Supplementary Information:**

The online version contains supplementary material available at 10.1186/s13048-026-01970-w.

## Introduction

With the improvement of socioeconomic conditions, the prevalence of mental health disorders has been steadily increasing, becoming a major global health threat. Mental health disorders, such as Attention-Deficit/Hyperactivity Disorder (ADHD), anxiety disorders, autism spectrum disorder, bipolar disorder, anorexia nervosa, depression, and Alzheimer’s disease, not only severely impact individuals’ mental health but may also have profound effects on women’s reproductive health [1]. In recent years, a growing body of research has indicated complex associations between mental health disorders and female reproductive system diseases, such as endometriosis, polycystic ovary syndrome (PCOS), uterine leiomyoma, and infertility [2]. However, the shared genetic mechanisms underlying these diseases remain poorly understood.

Both mental health disorders and female reproductive system diseases exhibit significant genetic components. For instance, the heritability estimates for bipolar disorder and depression are approximately 60–85% and 30–40%, respectively [3], while the heritability of PCOS and endometriosis is as high as 70% and 50% [4]. Additionally, family history studies suggest that these diseases tend to cluster within families, indicating that they may share certain genetic risk factors [5]. In recent years, genome-wide association studies (GWAS) have identified multiple genetic variants associated with both mental health disorders and female reproductive system diseases [6]. However, most of these studies have focused on single diseases, failing to comprehensively reveal the shared genetic architecture across these conditions.

Notably, there is a complex bidirectional relationship between mental health disorders and female reproductive system diseases. For example, individuals with depression and anxiety disorders are more likely to develop PCOS and infertility [7], while patients with endometriosis often experience comorbid anxiety and depressive symptoms [8]. These observations suggest that mental health disorders and female reproductive system diseases may influence each other through shared biological mechanisms. For instance, dysregulation of the hypothalamic-pituitary-adrenal (HPA) axis, activation of inflammatory responses, and abnormalities in sex hormone levels may serve as key pathways linking these diseases [9]. Furthermore, genetic factors may exacerbate the risk of comorbidity by influencing these pathways [10] .

Although epidemiological and clinical studies have provided important insights into the associations between mental health disorders and female reproductive system diseases, most of these studies have not delved deeply into the underlying genetic mechanisms [4]. Therefore, systematically investigating the shared genetic risk factors between these diseases will not only help uncover their comorbid mechanisms but may also provide a theoretical foundation for developing new therapeutic strategies [3].

This study aims to comprehensively explore the shared genetic architecture between mental health disorders and female reproductive system diseases by integrating large-scale GWAS data and advanced genetic analysis methods. We will employ genetic correlation analysis, cross-trait GWAS meta-analysis, and Mendelian randomization (MR) to identify shared genetic variants and risk genes across these diseases [6]. Additionally, we will use functional annotation and pathway analysis to reveal the biological mechanisms underlying these shared genetic variants [7]. Through this research, we hope to provide new insights into the comorbid mechanisms of mental health disorders and female reproductive system diseases and lay the groundwork for future precision medicine studies [8].

## Methods

**Fig. 1:**
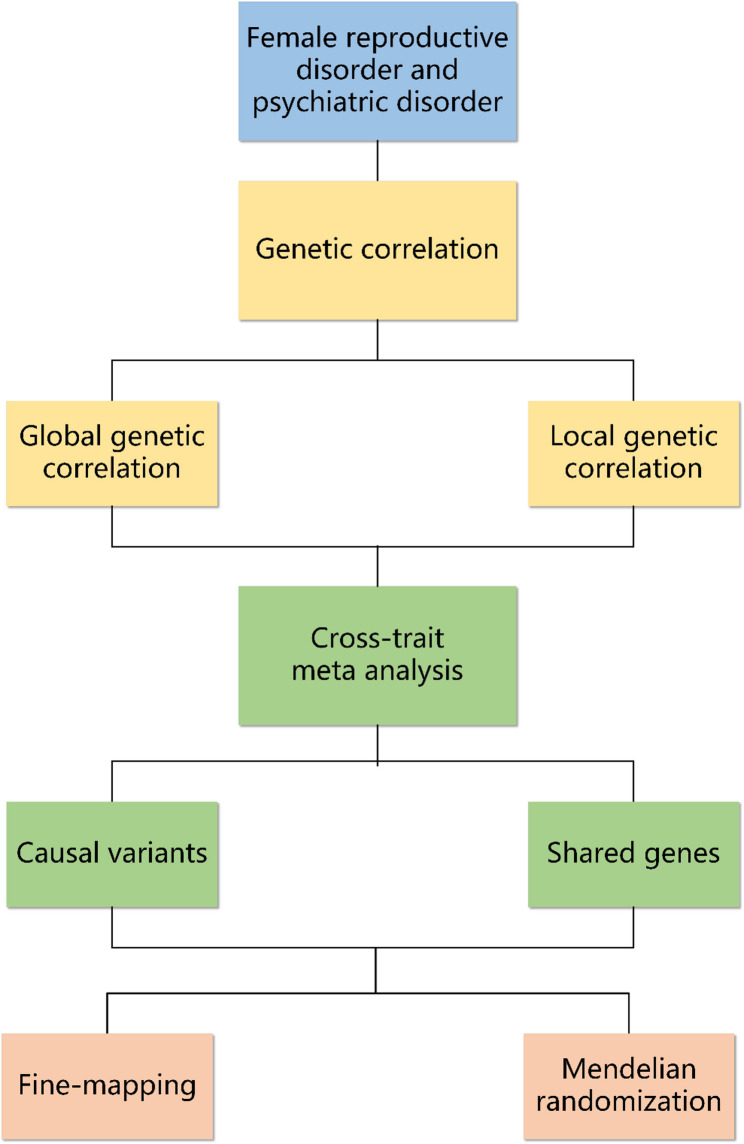
Flowchart of the study design. GWAS: Genome-wide association study; LDSC: Linkage disequilibrium score regression; HDL: High-Definition Likelihood; MTAG: Multi-trait Analysis of GWAS; CPASSOC: Cross Phenotype Association; MR: Mendelian randomization; SMR: Summary-data-based Mendelian randomization; MAGMA: Multi-marker Analysis of GenoMic Annotation; GCTA: Genome-wide Complex Trait Analysis

The GWAS data for psychiatric disorders were from the latest available data of the Psychiatric Genomics Consortium (PGC). The GWAS data for endometriosis, specifically from the European-ancestry subset, were derived from a meta-analysis of several large studies and are available in the GWAS Catalog (GCST90269970). This dataset explicitly excludes individuals of East Asian ancestry. The GWAS data for female infertility, polycystic ovary syndrome (PCOS), and uterine leiomyoma were from the FinnGen data version R12. Psychiatric disorders include major depressive disorder (MDD), schizophrenia, attention deficit hyperactivity disorder (ADHD), bipolar disorder (BD), autism spectrum disorder (ASD), anorexia nervosa (AN), and anxiety (Table 1). To evaluate potential sample overlap, we applied MR-Lap to the GWAS summary statistics for female reproductive disorders and psychiatric disorders. The intercept estimates were consistently below 0.1, suggesting that sample overlap was not a substantial concern in this analysis [11]. Genetic data processing was performed using the 1000 Genomes European population reference for standardization. Single nucleotide polymorphisms (SNPs) lacking rsIDs or possessing duplicate rsIDs were excluded. Missing data were imputed through a standard pipeline based on European-ancestry reference panels, which included quality control, phasing, and harmonization to the hg19 reference genome, followed by imputation using Minimac4 locally. Imputed variants were filtered based on a quality threshold of Rsq ≥ 0.7, and allelic dosage formats were retained for subsequent association analyses. A schematic of the research workflow is provided in Fig. 1. All statistical analyses in this study were carried out using the R package ‘DrugtargetMR’ [12].

**Table 1:** The detailed information of the source of GWAS

Diseases	Abbreviations	PMID	Year	N_cases	N_total	Ancestry
Endometriosis	En	36914876	2023	21,779	470,866	EUR
Female infertility	infertility	FinnGen	/	18,189	148,349	EUR
Polycystic ovary syndrome	PCOS	FinnGen	/	42,630	282,064	EUR
Leiomyoma	Leiomyoma	FinnGen	/	42,107	282,064	EUR
Major depressive disorder	MDD	30718901	2019	246,363	807,553	EUR
Bipolar disorder	BD	PGC	2024	156,643	2,956,105	EUR
Anxiety	/	26754954	2016	7016	21,761	EUR
Attention deficit hyperactivity disorder	ADHD	36702997	2023	38,691	225,534	EUR
Anorexia nervosa	AN	31308545	2019	16,992	72,517	EUR
Schizophrenia	/	31740837	2019	33,640	77,096	EUR
Autism Spectrum Disorder	ASD	30804558	2019	18,381	46,350	EUR

### LDSC

We estimated the genome-wide genetic correlations between traits using linkage disequilibrium score regression (LDSC). This method leverages the effect size estimates of individual genetic variants from GWAS, accounting for the aggregated effects of all variants in linkage disequilibrium(LD) with each locus. LDSC incorporates an intercept adjustment during analysis to address sample overlap across studies. The resulting genetic correlation estimates (rg) range from − 1 to 1, where − 1 indicates a complete negative genetic correlation and 1 represents a complete positive genetic correlation. A false discovery rate (FDR) correction was applied, with the significance threshold set at *P* < 0.05 [13].

### HDL

High-Definition Likelihood Inference (HDL) is a likelihood-based method that estimates genetic correlations using GWAS summary statistics. By refining the modeling of linkage disequilibrium (LD) structure, HDL provides more precise genetic correlation estimates compared to conventional approaches such as LD score regression (LDSC), achieving approximately 60% lower variance in genetic correlation estimates. This method is particularly advantageous for large-scale genome-wide association study (GWAS) datasets. The genetic correlation estimates range from − 1 to 1, where − 1 denotes a complete negative genetic correlation and 1 indicates a complete positive genetic correlation. An FDR correction was applied, with the significance threshold set at *P* < 0.05 [13].

### Cross-trait meta-analysis

To identify shared pleiotropic loci between traits, we applied Multi-Trait Analysis of GWAS (MTAG) for cross-trait meta-analysis of GWAS summary statistics [14]. MTAG employs a generalized inverse-variance-weighted meta-analytic approach to analyze multiple traits while accounting for potential sample overlap across GWAS. Its core assumption is that all single-nucleotide variants (SNVs) share a common variance-covariance matrix for their effect sizes across traits. As originally described, MTAG is a consistent estimator that reduces genome-wide mean squared error in effect estimates compared to single-trait GWAS. Additionally, MTAG-derived association statistics provide enhanced statistical power with minimal inflation of the false discovery rate (FDR) for each analyzed trait.

Given potential violations of MTAG’s assumptions (i.e., homogeneity in SNV heritability per trait and uniform genetic covariance across traits), we performed sensitivity analyses using Cross-Phenotype Association Analysis (CPASSOC) [15]. This method enhances detection power for shared genetic architectures between traits through meta-analysis, facilitating identification of putative pleiotropic loci. CPASSOC generates two test statistics: SHom and SHet. SHom employs fixed-effects meta-analysis and represents the maximum of weighted sums of trait-specific genetic effects, but exhibits reduced power under cross-study heterogeneity—a common feature of multi-trait meta-analyses. SHet, an extension of SHom, accommodates heterogeneity in study design, environmental factors, population ancestry, or phenotype-specific effects, making it more robust for real-world applications. Consequently, we adopted SHet in our analyses.

### Fine-mapping credible set analysis

Based on a Bayesian fine-mapping approach implemented in FM-summary (https://github.com/hailianghuang/FM-summary) [16], we defined a 99% credible set of causal variants. For each shared SNP identified through cross-trait meta-analysis, variants located within ± 500 kb of the index SNP were extracted as input for FM-summary. The method applied a flat prior and estimated posterior inclusion probabilities (PIPs) for phenotype–variant associations via the steepest descent approximation. The 99% credible set was constructed by ranking SNPs in descending order of PIPs and cumulatively summing PIPs until the total reached at least 99%.

### ANNOVAR

ANNOVAR is an efficient genomic variant annotation tool primarily used for functional annotation and classification of SNVs, insertions/deletions (InDels), and structural variants detected in high-throughput sequencing data [17]. This tool maps variants to different functional regions of the genome (e.g., exons, introns, promoters, or non-coding regulatory regions) and predicts their potential biological impact. We used ANNOVAR for functional annotation of the variants identified by MTAG and CPASSOC.

### Gene-based association analysis

Relying solely on physical proximity to assign GWAS variants to genes is an oversimplification that may fail to account for pleiotropy. To address this, we employed three complementary methods—SMR, MAGMA, and GCTAfastBAT—to infer shared genes between psychiatric disorders and female reproductive system disease traits. Input files for all analyses were derived from the complete GWAS summary statistics generated by MTAG in the meta-analysis, with P-value thresholds adjusted via Bonferroni correction to ensure statistical significance.

SMR analysis combines GWAS and eQTL (expression quantitative trait loci) data [18] to identify genes linked to complex traits through pleiotropy or causality. We performed SMR using cis-eQTL summary statistics from eQTLGen (whole blood) and GTEx tissues. The HEIDI-outlier test further distinguished pleiotropy from linkage effects, retaining genes with PSMR passing Bonferroni correction and PHEIDI > 0.05.

MAGMA employs a multiple regression model to integrate LD across markers and detect multi-marker effects [19]. Using the 1000 Genomes Project (Phase III) European ancestry panel as the LD reference, we ran MAGMA to identify polygenic signals associated with both psychiatric and female reproductive system diseases.

GCTA-fastBAT is a fast gene-based association method that calculates aggregate association P values for SNP sets using GWAS summary statistics and LD correlations from reference samples [20]. We similarly applied the 1000 Genomes Project European panel as the LD reference to identify gene sets associated with both disease trait pairs.

Through these three complementary approaches, we aimed to uncover shared genetic mechanisms between psychiatric disorders and female reproductive system diseases, providing critical insights for functional studies and clinical interventions.

### MR

To explore potential causal relationships between psychiatric disorders and reproductive health diseases, we conducted bidirectional MR analyses.

Independent genetic instruments (single-nucleotide polymorphisms, SNPs) for each exposure were selected at a genome-wide significance threshold (*P* < 5 × 10^-8). To ensure independence, we performed clumping using a strict LD threshold of r^2 < 0.001 within a 10,000 kb window, based on the 1000 Genomes European reference panel. Palindromic SNPs with intermediate allele frequencies were removed, and we harmonized the exposure and outcome summary statistics to ensure effect alleles were aligned. The strength of the instrumental variables was assessed using the F-statistic; all variants included had F-statistics > 10, indicating a low risk of weak instrument bias.

We implemented several MR methods to assess causal relationships: inverse-variance-weighted (IVW), MR-Egger, weighted median, and maximum likelihood [21–24]. These approaches rely on different assumptions regarding horizontal pleiotropy. The IVW method, a standard MR technique, derives causal effect estimates directly from summary-level genetic association data. MR-Egger extends this framework by including an intercept term in its regression model, which allows for the detection and correction of directional (non-zero mean) uncorrelated pleiotropy. The weighted median method provides robustness against invalid instrumental variables by computing the weighted median of individual Wald ratio estimates. Finally, the maximum likelihood method offers an alternative, model-based approach to estimate the causal effect under specified likelihood assumptions. Among these, the IVW method served as the primary analysis due to its standard application in MR studies and superior statistical power under the valid instrumental variable assumptions. The results from MR-Egger, weighted median, and maximum likelihood methods were considered as complementary sensitivity analyses to assess the consistency and reliability of the primary IVW estimates. When only one valid instrumental variable was available, we applied the Wald ratio method to estimate the causal effect.

To assess the robustness of our findings and validate the key MR assumptions, we conducted several sensitivity analyses. We used the MR-Egger intercept test to evaluate directional horizontal pleiotropy. Heterogeneity among the causal estimates of individual SNPs was quantified using Cochran’s Q statistic in both the IVW and MR-Egger methods. To identify and correct for potential outliers due to pleiotropy, we applied the MR-PRESSO (Mendelian Randomization Pleiotropy RESidual Sum and Outlier) test and reported the MR-PRESSO causal estimates after the removal of outlier variants.

To account for multiple testing across the examined trait pairs, an FDR correction was applied. The significance threshold was set at FDR < 0.05. Associations surviving this correction were considered strong evidence for a causal relationship, while those with a nominal P-value (*P* < 0.05) but not surviving FDR correction are indicated as such.

## Results

### LDSC

**Fig. 2:**
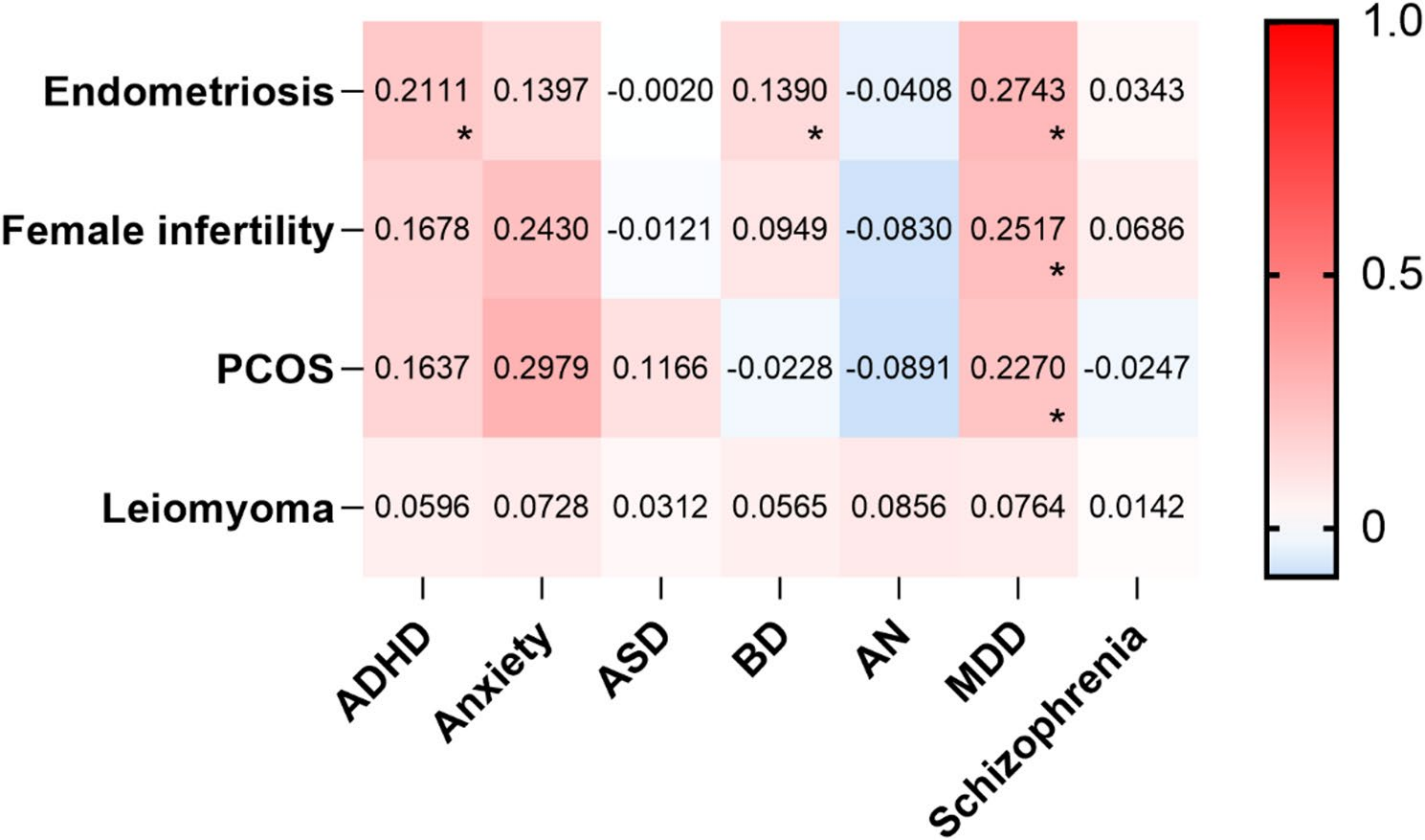
LDSC analysis between female reproductive disorders and psychiatric disorders. The heatmap presents the genetic correlation rg calculated in LDSC, with the color scale indicating the strength of the correlation, and the rg value displayed next to the heatmap. The * marks the statistical significance: *:P < 0.05 (FDR P value threshold). PCOS: polycystic ovary syndrome, ADHD: attention deficit hyperactivity disorder, ASD: autism spectrum disorder, BD: bipolar disorder, AN: anorexia nervosa, MDD: major depressive disorder

The LDSC analysis revealed that a total of 5 trait pairs remained significantly positively correlated after FDR correction, including endometriosis and ADHD (rg = 0.21; *P* = 1.40 × 10^− 5^); endometriosis and BD (rg = 0.14; *P* = 5.20 × 10^− 4^); endometriosis and MDD (rg = 0.27; *P* = 1.69 × 10^− 12^); female infertility and MDD (rg = 0.25; *P* = 3.05 × 10^− 6^); and PCOS and MDD (rg = 0.23; *P* = 9.38 × 10^− 5^). Results were shown in Fig. 2.

In the LDSC results, positive correlations were observed for female infertility and ADHD (rg = 0.17; *P* = 0.01), PCOS and ADHD (rg = 0.16; *P* = 0.03), and uterine leiomyoma and MDD (rg = 0.08; *P* = 9.38 × 10^− 5^), but no statistical significance was retained after FDR correction.

### HDL

**Fig. 3:**
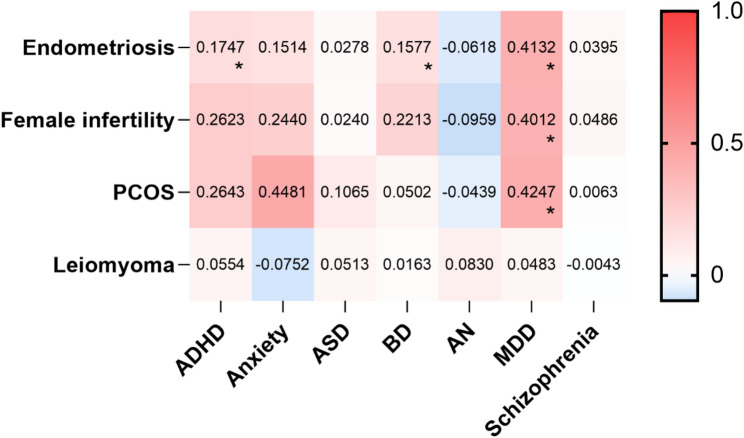
HDL analysis between female reproductive disorders and psychiatric disorders. The heatmap presents the genetic correlation rg calculated in HDL, with the color scale indicating the strength of the correlation, and the rg value displayed next to the heatmap. The * marks the statistical significance: *:P < 0.05 (FDR P value threshold). PCOS: polycystic ovary syndrome, ADHD: attention deficit hyperactivity disorder, ASD: autism spectrum disorder, BD: bipolar disorder, AN: anorexia nervosa, MDD: major depressive disorder

The HDL analysis revealed that a total of 5 trait pairs remained significantly positively correlated after FDR correction, including endometriosis and ADHD (rg = 0.17; *P* = 1.05 × 10^− 6^); endometriosis and BD (rg = 0.16; *P* = 1.75 × 10^− 2^); endometriosis and MDD (rg = 0.41; *P* = 4.72 × 10^− 12^); female infertility and MDD (rg = 0.40; *P* = 3.05 × 10^− 6^); and PCOS and MDD (rg = 0.42; *P* = 9.38 × 10^− 5^). Results were shown in Fig. 3.

While the HDL analysis showed positive correlations for female infertility and ADHD (rg = 0.26; *P* = 0.01), PCOS and ADHD (rg = 0.26; *P* = 0.03), and uterine leiomyoma and MDD (rg = 0.42; *P* = 9.38 × 10^− 5^), these associations did not retain statistical significance after FDR correction. A summary of all results from LDSC and HDL can be found in Supplementary Table S1.

### MTAG and CPASSOC

Cross-trait meta-analysis using MTAG (Multi-Trait Analysis of GWAS) [14] and CPASSOC (Cross-Phenotype Association Analysis) [15] identified 5 SNVs shared across 5 trait pairs (Table 2).

**Table 2:** Cross-trait meta-analysis between female reproductive disorders and psychiatric diseases

Cross-traits	SNP	CHR	BP	A1	A2	GWAS *P*-female reproductive disorders	GWAS *P*-psychiatric diseases	*P*-MTAG	*P*-CPASSOC
En_ADHD	rs9689096	6	34,221,115	C	A	3.71E-07	7.46E-04	4.96E-09	1.20E-08
En_ADHD	rs6650130	10	22,280,664	T	A	1.41E-06	3.85E-04	1.64E-08	1.76E-08
En_BD	rs11031047	11	30,315,976	T	C	8.61E-22	2.80E-04	1.70E-22	4.75E-25
En_MDD	rs633891	6	152,271,616	T	C	6.63E-09	3.99E-04	3.14E-11	1.43E-08
Infertility_MDD	rs633891	6	152,271,616	T	C	3.54E-09	3.99E-04	7.86E-11	5.95E-09
PCOS_MDD	rs10986104	9	123,786,056	T	C	4.27E-27	5.59E-06	1.13E-28	1.64E-27

*SNP* single nucleotide polymorphism, *CHR* chromosome, *BP* base pair position, *A1* effect allele, *A2* alternate allele, *MTAG* multi-trait analysis of GWAS, *CPASSOC* cross-phenotype association analysis, *En* endometriosis, *ADHD* attention deficit hyperactivity disorder, *BD* bipolar disorder, *MDD* major depressive disorder, *PCOS* polycystic ovary syndrome

Our analysis revealed that endometriosis and ADHD shared 2 loci (rs9689096 and rs6650130; PMTAG&CPASSOC < 5 × 10^− 8^, Psingle-trait < 1 × 10^− 3^); Endometriosis and MDD shared 1 locus (rs633891); Endometriosis and BD shared 1 locus (rs11031047); Female infertility and MDD shared 1 locus (rs633891); PCOS and MDD shared 1 locus (rs10986104).

### Fine-mapping

At the conclusion of the cross-trait meta-MTAG and CPASSOC analyses, we identified 6 independent pleiotropic SNPs across 5 pair traits. Based on these independent pleiotropic SNPs, genomic regions were selected for subsequent fine-mapping. Fine-mapping was then performed using the MTAG summary statistics of female reproductive disorders, yielding a set of high-confidence SNPs. An inCredible value of 1 indicates that the SNP is a credible causal variant within the 99% credible set, whereas a value of 0 signifies that the SNP falls outside this set. The probNorm value represents the normalized posterior probability for each SNP, indicating its relative likelihood of being a causal variant. A higher value corresponds to a greater probability that the SNP is causal. Apart from the shared SNPs between endometriosis and MDD, which are not credible causal variants, the SNPs for all other trait pairs are credible causal variants. Results were shown in Table 3.

**Table 3:** Results of finemapping

Cross-traits	SNP	CHR	BP	A1	A2	GWAS *P*-female reproductive disorders	GWAS *P*-psychiatric diseases	*P*-MTAG	*P*-CPASSOC	inCredible	probNorm
En_ADHD	rs9689096	6	34,221,115	C	A	3.71E-07	7.46E-04	4.96E-09	1.20E-08	1	0.21
En_ADHD	rs6650130	10	22,280,664	T	A	1.41E-06	3.85E-04	1.64E-08	1.76E-08	1	0.14
En_BD	rs11031047	11	30,315,976	T	C	8.61E-22	2.80E-04	1.70E-22	4.75E-25	1	0.25
Infertility_MDD	rs633891	6	152,271,616	T	C	3.54E-09	3.99E-04	7.86E-11	5.95E-09	1	0.99
PCOS_MDD	rs10986104	9	123,786,056	T	C	4.27E-27	5.59E-06	1.13E-28	1.64E-27	1	0.44

*SNP* single nucleotide polymorphism, *CHR* chromosome, *BP* base pair position, *A1* effect allele, *A2* alternate allele, *MTAG* multi-trait analysis of GWAS, *CPASSOC* cross-phenotype association analysis, *En* endometriosis, *ADHD* attention deficit hyperactivity disorder, *BD* bipolar disorder, *MDD* major depressive disorder, *PCOS* polycystic ovary syndrome

### ANNOVAR

Genetic variants identified through association analyses were functionally annotated using ANNOVAR, a high-performance tool for characterizing sequence variations. The shared SNPs between endometriosis and ADHD are rs9689096 and rs6650130, which were annotated to the genes *SMIM29* and *DNAJC1* respectively. The shared SNP between endometriosis and BD is rs11031047, annotated to the shared gene *ARL14EP.* The shared SNP between female infertility and MDD is rs633891, annotated to the shared gene *ESR1*. The shared SNP between PCOS and MDD is rs10986104, annotated to the shared gene *C5*. Results were shown in Table 4. In the main text, we will only present pleiotropic SNPs along with their fine-mapping and ANNOVAR annotation results for trait pairs that exhibit genetic correlation. The remaining pleiotropic SNPs and their corresponding fine-mapping and ANNOVAR results for all other trait pairs will be provided in Supplementary Table S2 for reference.

**Table 4:** Results of ANNOVAR

Cross-traits	SNP	CHR	BP	A1	A2	GWAS *P*-female reproductive disorders	GWAS *P*-psychiatric diseases	*P*-MTAG	*P*-CPASSOC	Consequence	NearestGene
En_ADHD	rs9689096	6	34,221,115	C	A	3.71E-07	7.46E-04	4.96E-09	1.20E-08	intergenic	SMIM29
En_ADHD	rs6650130	10	22,280,664	T	A	1.41E-06	3.85E-04	1.64E-08	1.76E-08	intronic	DNAJC1
En_BD	rs11031047	11	30,315,976	T	C	8.61E-22	2.80E-04	1.70E-22	4.75E-25	intergenic	ARL14EP
Infertility_MDD	rs633891	6	152,271,616	T	C	3.54E-09	3.99E-04	7.86E-11	5.95E-09	intronic	ESR1
PCOS_MDD	rs10986104	9	123,786,056	T	C	4.27E-27	5.59E-06	1.13E-28	1.64E-27	intronic	C5

*SNP* single nucleotide polymorphism, *CHR* chromosome, *BP* base pair position, *A1 *effect allele, *A2 *alternate allele, *MTAG* multi-trait analysis of GWAS, *CPASSOC* cross-phenotype association analysis, *En* endometriosis, *ADHD* attention deficit hyperactivity disorder, *BD* bipolar disorder, *MDD* major depressive disorder, *PCOS* polycystic ovary syndrome

### Shared genes

Previous gene annotation of GWAS variants based solely on genomic proximity risks oversimplification and may fail to account for pleiotropy. To address this, we integrated three methods—SMR [18], MAGMA [19], and GCTA-fastBAT [20]—to infer shared genes. SMR relies on expression quantitative trait loci (eQTL), while the latter two primarily assess gene-based burden testing through proximity. Genes consistently supported by all three methods were defined as disease-associated. Our results indicate that the shared gene *ARL14EP* between endometriosis and bipolar disorder was consistently validated by all three gene analysis approaches. For all other trait pairs, however, no shared genes were jointly confirmed by multiple gene analysis methods. (Supplementary Table S3).

### Mendelian randomization

**Fig. 4:**
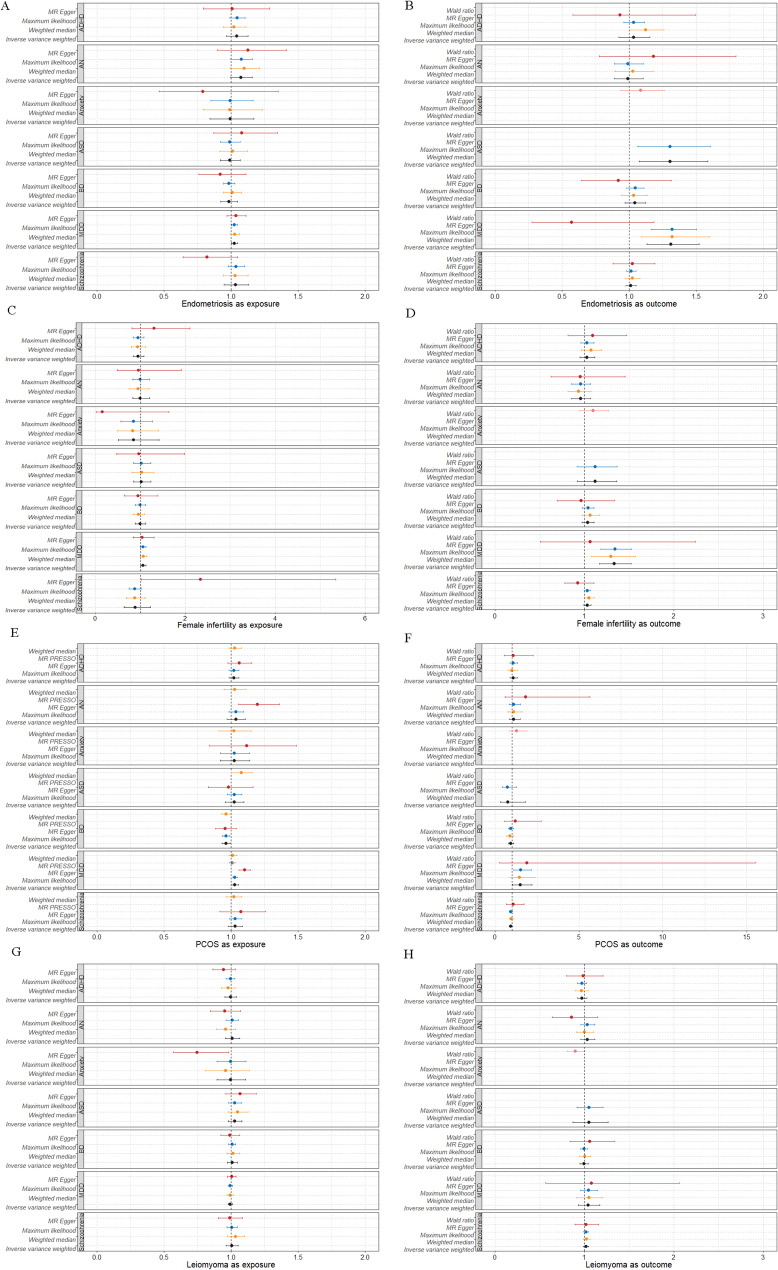
Causal inference between female reproductive disorders and psychiatric disorders. Causal inference by two-sample Mendelian Randomization with five methods. Odds ratios are shown as dots, the color bars present +/− 95% confidence intervals, and P values are depicted above the bars. ADHD: attention deficit hyperactivity disorder, AN: anorexia nervosa, ASD: autism spectrum disorder, BD: bipolar disorder, MDD: major depressive disorder, PCOS: polycystic ovary syndrome. All reported P values are two-sided, unless stated otherwise (**A**) Mendelian randomization estimates of the causal effects of endometriosis on psychiatric disorders. (**B**) Mendelian randomization estimates of the causal effects of psychiatric disorders on endometriosis. (**C**) Mendelian randomization estimates of the causal effects of female infertility on psychiatric disorders. (**D**) Mendelian randomization estimates of the causal effects of psychiatric disorders on female infertility. (**E**) Mendelian randomization estimates of the causal effects of PCOS on psychiatric disorders. (**F**) Mendelian randomization estimates of the causal effects of psychiatric disorders on PCOS. (**G**) Mendelian randomization estimates of the causal effects of leiomyoma on psychiatric disorders. (**H**) Mendelian randomization estimates of the causal effects of psychiatric disorders on leiomyoma

Bidirectional MR analyses using multiple models revealed causal effects of MDD on endometriosis (OR = 1.31, P^IVW−FDR^ = 1.55 × 10⁻^2^), female infertility (OR = 1.33, P^IVW−FDR^ = 1.93 × 10^⁻3^) (Fig. 4). The complete results of all MR analyses, along with the results of sensitivity analyses, are provided in Supplementary Tables S4 and S5.

## Discussion

This study presents the first comprehensive investigation into the shared genetic architecture between major female reproductive disorders—endometriosis, infertility, PCOS, and uterine leiomyoma—and a spectrum of psychiatric conditions. By integrating global/local genetic correlation, cross-trait meta-analysis, fine-mapping, multi-method gene prioritization, and bidirectional MR, we not only employed MR to substantiate a potential causal association between MDD and both endometriosis and infertility, but also identified multiple specific genetic loci and genes with cross-disorder effects such as *ESR1* and *ARL14EP*.

Previous genetic studies have examined single reproductive disorder-psychiatric condition pairs, such as endometriosis with depression and anxiety, PCOS with multiple psychiatric disorders, uterine leiomyoma with psychological distress, and reproductive timing traits with psychiatric phenotypes. However, these studies have yielded inconsistent findings and have not comprehensively revealed the shared genetic architecture across multiple reproductive disorders and psychiatric conditions [25–28]. This study is the first large-scale cross-trait analysis exploring the shared genetic basis between psychiatric disorders and female reproductive system diseases. Using LDSC and HDL methods, we identified significant global genetic correlations between endometriosis and ADHD, as well as among endometriosis, female infertility, PCOS, and MDD. Extensive existing evidence indicates an association between endometriosis and depression. Epidemiological studies have reported their frequent co-occurrence [29, 30]. The findings of the current study are consistent with recent research by Rostvall et al., which identified a genome-wide genetic correlation between endometriosis and ADHD, as well as bipolar disorder [31]. A 2024 study reported familial co-aggregation between MDD and PCOS [32]. In a study sample of 79 patients with endometriosis, Skegro et al. observed a moderate correlation between depression and infertility [33]. Our study meaningfully advance current understanding by demonstrating genome-wide genetic correlations, thereby providing robust evidence for a shared biological basis underlying the clinically observed comorbidities between these conditions.

Pleiotropic SNPs tested by CPASSOC and MTAG were annotated via ANNOVAR, revealing five shared loci between female reproductive diseases and psychiatric disorders—two linked to ADHD, one to BD and three to MDD. Subsequent fine mapping and functional annotation further identified genes *ESR1* and *ARL14EP* shared across two phenotype pairs.

Our integrated genetic analysis reveals a robust pleiotropic association between the *ARL14EP* locus and both BD and endometriosis. *ARL14EP* functions as a key epigenetic regulator critical for the development of callosal connectivity in the brain. By modulating the expression of genes involved in axonal development, it influences the establishment of interhemispheric neural connections [34, 35]. The corpus callosum serves as a core structure for integrating information between cerebral hemispheres and coordinating emotion with cognition. Microstructural abnormalities of the corpus callosum are closely linked to emotional dysregulation and reduced cognitive flexibility in BD. *ARL14EP* likely acts as a fundamental chromatin-regulatory protein across multiple cell types, including neurons, immune cells, and endometrial cells. Its functional impairment may contribute to a chronic low-grade inflammatory state—a shared feature of both BD and endometriosis. Thus, *ARL14EP* could represent a key molecular node linking dysregulation of the brain–immune–endocrine axis.

The co-association with endometriosis suggests that the role of *ARL14EP* as a chromatin regulator extends beyond the central nervous system. This locus may act as a **“**systemic pleiotropic node**”**, simultaneously affecting tissue integrity and inflammatory responses in both the brain and the reproductive system. This observation aligns with the growing recognition that BD and endometriosis share overlapping immune–endocrine disturbances [36, 37]. Therefore, *ARL14EP* is not merely a risk gene for two distinct disorders, but rather a key component within a shared pathophysiological network that connects neurodevelopment, hormonal signaling, and immune function.

The observed genetic overlap between endometriosis and ADHD suggests the involvement of common biological pathways, such as hormonal regulation and neurodevelopmental processes. We identified a shared SNP (rs9689096) annotated to the *SMIM29* gene. *SMIM29* (Small Integral Membrane Protein 29) is a small transmembrane protein localized to the cell membrane. Although its molecular function is not completely clarified, existing evidence suggests potential roles in cell signaling, membrane receptor regulation, and immune-inflammatory responses. *SMIM29* exhibits broad tissue expression, including the brain, ovaries, and endometrium, implying its dual function to neurodevelopment and reproductive physiology. Notably, functional studies of *SMIM29* have focused solely on respiratory system, leaving its mechanistic contributions to neurological or female reproductive disorders entirely unexplored. In the future, large-scale GWAS integrative analyses, functional validations, or cross-disease phenotype studies are needed to discover whether SMIM29 mediates ADHD-endometriosis comorbidity through pleiotropic mechanisms.

Our study identified a significant positive genetic correlation between endometriosis and MDD, suggesting a shared biological basis at the genetic level. Cross-trait meta-analysis revealed a shared single nucleotide polymorphism (rs663891) annotated to the *ESR1* gene. Although rs663891 was not identified as a highly confident causal variant in the present study and was thus excluded from subsequent gene-level analyses, the role of *ESR1* in the pathogenesis of both endometriosis and depression has been extensively documented in previous literature. The *ESR1* gene encodes estrogen receptor alpha (ERα), a ligand-activated transcription factor with canonical structural domains: an N-terminal ligand-independent transcription-activation domain, a central DNA-binding domain, a hinge region, and a C-terminal ligand-dependent transcription-activation domain. ERα is localized to the nucleus and forms homodimers or heterodimers with estrogen receptor beta (ERβ) to regulate estrogen-responsive genes, governing growth, metabolism, sexual development, and pregnancy, with expression extending to non-reproductive tissues. McGrath et al. revealed molecular biology of commonality between endometriosis and depression via shared genes and pathways [38]. Furthermore, Proestling et al. demonstrated that *ESR1* polymorphisms correlated with upregulated expression in young patients, which might increase the risk of severe endometriosis [39]. In female MDD pathogenesis, *ESR1* plays an important role through polymorphisms, expression dynamics, and pathway crosstalk. Ryan et al. reported that two *ESR1* polymorphic variants were significantly associated with recurrent MDD risk: Women carrying the rs9340799 G allele had a 1.6-fold increased lifetime risk of MDD (*p* = 0.009) [40]. To our knowledge, this study is the first to provide genetic epidemiology data nominating the *ESR1* locus as a potential shared genetic link for the endometriosis-MDD comorbidity. Integrating this evidence, we propose a potential mechanistic framework centered on estrogen signaling to explain this comorbidity: Estrogen fluctuations could serve as a common biological trigger, whereby dysregulated signaling via ERα might concurrently promote ectopic endometrial cell survival and proliferation, and modulate neurotransmitter systems (e.g., serotonin, dopamine) and stress-response circuits (e.g., HPA axis) in the brain, leading to co-occurring pathology.

Various studies have indicated that specific variants of the *ESR1* gene (such as rs9340799 and rs2234693) had potential associations with female infertility. One study demonstrated a significant correlation between *ESR1* gene rs9340799 polymorphism and female infertility, with these variants potentially affecting embryo implantation. Research by Qin et al. found that rs2234693 and rs9340799 polymorphisms of the *ESR1* gene were significantly associated with natural menopause age and premature ovarian failure, suggesting these genetic variations may influence ovarian function in females, thereby affecting fertility [41]. Also, our finding is particularly noteworthy as it complements the recent identification by Koller et al. of *DGKB* rs12666606 as a pleiotropic variant between endometriosis and depression [27]. Different studies have consistently concluded that abnormalities in the estrogen signaling pathway caused by *ESR1* gene variations may be responsible for both MDD and female infertility, suggesting a possible comorbidity mechanism between these two diseases [42]. However, due to factors such as genetic heterogeneity and sample selection limitations, current research findings still require further validation and refinement.

This study also identified a potential association between PCOS and MDD at the genetic locus rs10986104, which is annotated to the *C5* gene. The complement system plays an important role in both neuroinflammation and endocrine regulation, which theoretically supports its role as a potential mediator gene for the co-morbidity between PCOS and MDD [43]. However, none of the three genetic analysis methods showed that *C5* was a significantly shared gene between the two traits, a contradiction that may stem from the following reasons: first, rs10986104 may affect the expression of distal genes, rather than *C5* itself, through long-distance regulation or epigenetic modification, resulting in the failure of gene-level-based analysis methods to capture its effects [44]. For example, the locus may be located in an enhancer region that regulates other genes associated with inflammation or hormone metabolism, thus indirectly affecting both diseases [45]. Second, both PCOS and MDD are polygenic disorders in which the contribution of individual loci may be insufficient and the statistical power of traditional genetic analysis methods is insufficient to detect their weak but real effects [3]. Future studies are needed to validate the regulatory targets of rs10986104 in combination with chromatin interactions data and to resolve the cell type-specific expression pattern of *C5* in ovarian and brain tissues using single-cell sequencing [46]. In addition, pharmacological interventions targeting the complement pathway such as *C5* inhibitors may provide an experimental basis for exploring mechanisms of co-morbidities [47].

Our finding contrasts with a previous study by Jiang et al. (2021), which reported no significant genetic correlation between PCOS and depression (rg = 0.09, *P* = 0.06) [28]. The discrepancy may be attributable to differences in GWAS sample sizes (our MDD GWAS included 246,363 cases compared to their 170,756 cases), phenotypic heterogeneity in PCOS diagnosis, or methodological differences in genetic correlation estimation. However, the consistency of our findings across multiple analytical methods (LDSC and HDL) and the replication across independent cohorts support the robustness of the genetic relationship between PCOS and MDD. Moreover, our identification of specific shared genetic loci (rs10986104 in *C5*) provides molecular evidence for pleiotropy that was not available in previous analyses.

The identification of shared genetic variants and pathways between endometriosis and BD, endometriosis and ADHD, and infertility and MDD provides a foundation for future research into the biological mechanisms underlying these comorbidities. Functional studies, such as transcriptomic and epigenomic analyses, are needed to validate the role of identified genes and pathways. Additionally, these findings have clinical implications, suggesting that personalized treatment strategies targeting shared biological pathways may improve outcomes for individuals with these comorbid conditions.

In conclusion, this genetic study reveals significant shared heritability between female reproductive disorders and psychiatric conditions. We identified robust genetic correlations, particularly linking endometriosis with ADHD, BD and MDD. A key robust finding is the consistent validation of *ARL14EP* as a shared risk gene for endometriosis and BD across three complementary methods (SMR, MAGMA, and GCTAfastBAT). MR further supports a potential causal role of MDD in endometriosis and infertility. These results underscore a common genetic basis for the observed comorbidities. Future research should focus on elucidating the biological mechanisms of *ARL14EP* and other identified candidate genes, as well as investigating their potential as therapeutic targets or biomarkers for early detection in high-risk populations.

## Supplementary Information

Supplementary Material 1.

Supplementary Material 2.

Supplementary Material 3.

Supplementary Material 4.

Supplementary Material 5.

## Data Availability

The data analyzed in this study were sourced from publicly available databases, specifically the IEU Open GWAS Project ( [https://gwas.mrcieu.ac.uk/datasets](https:/gwas.mrcieu.ac.uk/datasets) ), the PGC (https://pgc.unc.edu/for-researchers/download-results/), and the FinnGen Biobank.
