# Supplementary material for: Deciphering the shared genetic architecture between female reproductive disorders and psychiatric disorders
Source: J Ovarian Res. 2026 Jan 23;19:62. doi: 10.1186/s13048-026-01970-w (PMC12910984; doi:10.1186/s13048-026-01970-w)
Supplement: Supplementary file 1 — Supplementary Material 1. [file 13048_2026_1970_MOESM1_ESM.docx]

**Supplementary Table S1.** The source of GWAS and genome-wide genetic correlation between reproductive disorders and psychiatric disorders using LDSC and HDL

| Trait1 | Trait2 | LDSC | | | | HDL | | | |
| --- | --- | --- | --- | --- | --- | --- | --- | --- | --- |
|  |  | rg | rg_se | rg_p | rg_p* | rg | rg_se | rg_p | rg_p* |
| En | ADHD | 2.11E-01 | 4.58E-02 | 4.01E-06 | 1.40E-05 | 1.75E-01 | 3.43E-02 | 3.51E-07 | 1.05E-06 |
| En | Anxiety | 1.40E-01 | 1.25E-01 | 2.62E-01 | 3.82E-01 | 1.51E-01 | 8.49E-02 | 7.43E-02 | 1 |
| En | ASD | -2.04E-03 | 6.77E-02 | 9.76E-01 | 9.76E-01 | 2.78E-02 | 4.15E-02 | 5.02E-01 | 5.02E-01 |
| En | BD | 1.39E-01 | 3.76E-02 | 2.23E-04 | 5.20E-04 | 1.58E-01 | 6.02E-02 | 8.74E-03 | 1.75E-02 |
| En | AN | -4.08E-02 | 4.08E-02 | 3.18E-01 | 3.82E-01 | -6.18E-02 | 3.76E-02 | 1.00E-01 | 1.20E-01 |
| En | MDD | 2.74E-01 | 3.74E-02 | 2.41E-13 | 1.69E-12 | 4.13E-01 | 5.77E-02 | 7.86E-13 | 4.72E-12 |
| En | Schizophrenia | 3.43E-02 | 3.50E-02 | 3.28E-01 | 3.82E-01 | 3.95E-02 | 2.25E-02 | 7.95E-02 | 1.19E-01 |
| Infertility | ADHD | 1.68E-01 | 4.78E-02 | 4.52E-04 | 1.27E-02 | 2.62E-01 | 6.26E-02 | 2.77E-05 | 1.27E-02 |
| Infertility | Anxiety | 2.43E-01 | 1.50E-01 | 1.06E-01 | 1 | 2.44E-01 | 1.15E-01 | 3.45E-02 | 1 |
| Infertility | ASD | -1.21E-02 | 6.91E-02 | 8.61E-01 | 1 | 2.40E-02 | 7.91E-02 | 7.61E-01 | 1 |
| Infertility | BD | 9.49E-02 | 4.17E-02 | 2.28E-02 | 6.38E-01 | 2.21E-01 | 7.69E-02 | 4.00E-03 | 6.38E-01 |
| Infertility | AN | -8.30E-02 | 5.16E-02 | 1.07E-01 | 1 | -9.59E-02 | 6.35E-02 | 1.31E-01 | 1 |
| Infertility | MDD | 2.52E-01 | 4.74E-02 | 1.09E-07 | 3.05E-06 | 4.01E-01 | 9.41E-02 | 2.00E-05 | 3.05E-06 |
| Infertility | Schizophrenia | 6.86E-02 | 4.00E-02 | 8.63E-02 | 1 | 4.86E-02 | 4.76E-02 | 3.08E-01 | 1 |
| PCOS | ADHD | 1.64E-01 | 5.06E-02 | 1.21E-03 | 3.38E-02 | 2.64E-01 | 1.01E-01 | 8.83E-03 | 3.38E-02 |
| PCOS | Anxiety | 2.98E-01 | 1.63E-01 | 6.68E-02 | 1 | 4.48E-01 | 1.83E-01 | 1.43E-02 | 1 |
| PCOS | ASD | 1.17E-01 | 6.23E-02 | 6.11E-02 | 1 | 1.07E-01 | 9.55E-02 | 2.65E-01 | 1 |
| PCOS | BD | -2.28E-02 | 5.36E-02 | 6.71E-01 | 1 | 5.02E-02 | 1.14E-01 | 6.60E-01 | 1 |
| PCOS | AN | -8.91E-02 | 6.39E-02 | 1.63E-01 | 1 | -4.39E-02 | 1.03E-01 | 6.69E-01 | 1 |
| PCOS | MDD | 2.27E-01 | 4.88E-02 | 3.35E-06 | 9.38E-05 | 4.25E-01 | 1.19E-01 | 3.45E-04 | 9.38E-05 |
| PCOS | Schizophrenia | -2.47E-02 | 4.82E-02 | 6.08E-01 | 1 | 6.30E-03 | 7.26E-02 | 9.31E-01 | 1 |
| Leiomyoma | ADHD | 5.96E-02 | 2.97E-02 | 4.50E-02 | 1 | 5.54E-02 | 3.48E-02 | 1.12E-01 | 1 |
| Leiomyoma | Anxiety | 7.28E-02 | 9.00E-02 | 4.19E-01 | 1 | -7.52E-02 | 6.14E-02 | 2.21E-01 | 1 |
| Leiomyoma | ASD | 3.12E-02 | 3.89E-02 | 4.22E-01 | 1 | 5.13E-02 | 3.71E-02 | 1.67E-01 | 1 |
| Leiomyoma | BD | 5.65E-02 | 2.79E-02 | 4.30E-02 | 1 | 1.63E-02 | 4.16E-02 | 6.95E-01 | 1 |
| Leiomyoma | AN | 8.56E-02 | 3.68E-02 | 2.02E-02 | 5.64E-01 | 8.30E-02 | 3.80E-02 | 2.92E-02 | 5.64E-01 |
| Leiomyoma | MDD | 7.64E-02 | 2.86E-02 | 7.46E-03 | 2.09E-01 | 4.83E-02 | 4.83E-02 | 3.17E-01 | 2.09E-01 |
| Leiomyoma | Schizophrenia | 1.42E-02 | 2.44E-02 | 5.61E-01 | 1 | -4.30E-03 | 3.01E-02 | 8.86E-01 | 1 |
| GWAS: Genome-Wide association study, LDSC: Linkage disequilibrium score regression, HDL: High-Definition likelihood, rg: genetic correlation, h2: heritability, se: standard error, En: Endometriosis, PCOS: Polycystic ovary syndrome, ADHD: Attention deficit hyperactivity disorder, ASD: Autism spectrum disorder, BD: Dipolar disorder, AN: Anorexia nervosa, MDD: Major depressive disorder, *represents through false discovery rate(FDR) correction. | | | | | | | | | |
